# Short chain fatty acids-producing and mucin-degrading intestinal bacteria predict the progression of early Parkinson’s disease

**DOI:** 10.1038/s41531-022-00328-5

**Published:** 2022-06-01

**Authors:** Hiroshi Nishiwaki, Mikako Ito, Tomonari Hamaguchi, Tetsuya Maeda, Kenichi Kashihara, Yoshio Tsuboi, Jun Ueyama, Takumi Yoshida, Hiroyuki Hanada, Ichiro Takeuchi, Masahisa Katsuno, Masaaki Hirayama, Kinji Ohno

**Affiliations:** 1 Division of Neurogenetics, Center for Neurological Diseases and Cancer, Nagoya University Graduate School of Medicine, Nagoya, Japan; 2 Division of Neurology and Gerontology, Department of Internal Medicine, School of Medicine, Iwate Medical University, Iwate, Japan; 3 Department of Neurology, Okayama Kyokuto Hospital, Okayama, Japan; 4 Department of Neurology, Fukuoka University, Fukuoka, Japan; 5 Department of Pathophysiological Laboratory Sciences, Nagoya University Graduate School of Medicine, Nagoya, Japan; 6 Department of Computer Science, Nagoya Institute of Technology, Nagoya, Japan; 7 Center for Advanced Intelligence Project, RIKEN, Tokyo, Japan; 8 Department of Neurology, Nagoya University Graduate School of Medicine, Nagoya, Japan

**Keywords:** Parkinson's disease, Prognostic markers

## Abstract

To elucidate the relevance of gut dysbiosis in Parkinson’s disease (PD) in disease progression, we made random forest models to predict the progression of PD in two years by gut microbiota in 165 PD patients. The area under the receiver operating characteristic curves (AUROCs) of gut microbiota-based models for Hoehn & Yahr (HY) stages 1 and 2 were 0.799 and 0.705, respectively. Similarly, gut microbiota predicted the progression of Movement Disorder Society-Unified Parkinson’s Disease Rating Scale (MDS-UPDRS) III scores in an early stage of PD with AUROC = 0.728. Decreases of short-chain fatty acid-producing genera, *Fusicatenibacter*, *Faecalibacterium*, and *Blautia*, as well as an increase of mucin-degrading genus *Akkermansia*, predicted accelerated disease progression. The four genera remained unchanged in two years in PD, indicating that the taxonomic changes were not the consequences of disease progression. PD patients with marked gut dysbiosis may thus be destined to progress faster than those without gut dysbiosis.

## Introduction

Parkinson’s disease (PD) is a long-term neurodegenerative disease that exhibits not only motor symptoms but also non-motor symptoms^1^. PD is attributed to the loss of dopaminergic neurons in the substantia nigra. The loss is caused by abnormal aggregation of α-synuclein fibrils (Lewy bodies) in the neuronal cells. Lewy bodies also exist in the lower brainstem and the cerebral cortex^2^, the olfactory bulb^3^, the salivary glands^4^, the skin^5^, the autonomic nervous system^6^, and the intestine^4,7,8^. The Braak’s breakthrough paper and the following studies indicated that abnormal aggregation of α-synuclein fibrils starts in the intestinal nerve plexus and gradually moves up to the substantia nigra^2,3,9,10^. Constipation, idiopathic rapid eye movement sleep behavior disorder (iRBD), and depression can be frequently observed about 20, 10, and 5 years before the development of motor symptoms in PD^1^, which is consistent with the Braak’s hypothesis. In rodent models, gastrointestinal injection of pathogenic α-synuclein causes propagation of α-synuclein aggregates to brain via the vagus nerve^11–14^ and neurodegeneration of the substantia nigra^14^. In common marmoset, pathologic α-synuclein transmits within the brain and can be neurotoxic^15^. Similarly, in baboon monkey, pathogenic α-synuclein transmits bidirectionally between the enteric and the central nervous systems even in the absence of α-synuclein pathology in the vagus nerve^16^.

Epidemiological studies indicate that older age, male, cognitive impairment, and postural instability/gait-dominant type of PD are predictive of rapid progression of PD^17–21^. A single machine-learning model to predict the progression of PD has been reported, and will be addressed in detail in the discussion^22^.

As far as we know, 19 studies in PD^23–41^ and one study in iRBD^42^ have been reported on gut microbiota. Another study analyzed gut microbiota in both PD and iRBD^43^. We showed by meta-analysis of gut microbiota in five countries that genus *Akkermansia* was increased and genera *Roseburia* and *Faecalibacterium* were decreased in PD^38^. In contrast, in a meta-analysis of iRBD in Germany and Japan, genus *Akkermansia* was increased, whereas genera *Roseburia* and *Faecalibacterium* were not decreased^42^. *Akkermansia* degrades the intestinal mucin layer,^44,45^ and is predicted to increase the intestinal permeability, which has been reported in PD by us^23^ and others^46^. Genera *Roseburia* and *Faecalibacterium* produce short chain fatty acids (SCFAs). Decreased SCFAs are potentially linked to activated neuroinflammations in PD^47,48^. In Finland, genus *Prevotella* was decreased in PD, and PD patients with lower relative abundance of genus *Prevotella* tended to progress faster in two years^24,41^. In our meta-analysis of PD in five countries, however, significant decrease of genus *Prevotella* was observed in Finland, but not in United States, Russia, Germany, or Japan^38^. Similarly, genus *Prevotella* was not decreased in iRBD in Germany or Japan^42,43^. We here examined whether gut microbiota could predict the progression of PD in two years.

## Results

### Clinical features of PD patients at years 0 and 2

We collated clinical features of PD patients at years 0 and 2 (Supplementary Table S1). Total MDS-UPDRS, HY stage, levodopa/carbidopa dosage, walking and balance (MDS-UPDRS 2.12), freezing (MDS-UPDRS 2.13), gait (MDS-UPDRS 3.10), freezing of gait (MDS-UPDRS 3.11), and postural stability (MDS-UPDRS 3.12) were significantly different between years 0 and 2.

We also collated clinical features (Table 1), as well as clinical features in the stable and deteriorated groups (Supplementary Table S2), for each HY stage at year 0. Fifteen out of 35 features were significantly different between HY stages 1, 2, and 3. The significant difference was observed only in reasonable features like age, disease duration, and MDS-UPDRS scores.

**Table 1:** Clinical and demographic features of PD patients at Hoehn & Yahr stages 1, 2, and 3 at year 0 used to generate random forest models.

	Hoehn & Yahr 1 (*n* = 24)^a^	Hoehn & Yahr 2 (*n* = 85)^a^	Hoehn & Yahr 3 (*n* = 56)^a^	*P*-value^b^
# Stable group	12 (50.0%)	56 (65.9%)	44 (78.6%)	^*^0.035
Age (years)	66.3 ± 9.0	65.9 ± 9.3	70.4 ± 6.7	^*^9.2E-3
# Females	19 (79.2%)	46 (54.1%)	31 (55.4%)	0.075
Body mass index (BMI)	20.9 ± 2.8	22.2 ± 3.0	21.1 ± 3.2	0.054
# Constipation (≤twice a week)	7 (29.2%)	26 (30.6%)	25 (44.6%)	0.19
Stool frequency/week	4.5 ± 2.3	5.9 ± 5.8	4.0 ± 3.1	0.16
Disease duration (years)	3.9 ± 3.2	5.2 ± 4.0	10.0 ± 5.7	^*^2.4E-6
Total MDS-UPDRS	31.5 ± 11.9	43.2 ± 15.9	58.5 ± 20.8	^*^1.0E-9
	(range 11–68)	(range 12–92)	(range 17–115)	
MDS-UPDRS III	16.1 ± 7.6	23.0 ± 10.5	31.4 ± 12.1	^*^3.6E-8
	(range 4–42)	(range 4–56)	(range 5–61)	
# Proton pump inhibitor	2 (8.3%)	13 (15.3%)	7 (12.5%)	0.74
# H_2_ blocker	1 (4.2%)	2 (2.4%)	4 (7.1%)	0.36
# Antihyperlipidemic drug	2 (8.3%)	17 (20.0%)	8 (14.3%)	0.38
# Angiotensin II receptor blocker	1 (4.2%)	8 (9.4%)	11 (19.6%)	0.12
# Calcium channel blocker	3 (12.5%)	14 (16.5%)	11 (19.6%)	0.75
Levodopa/Carbidopa (mg)	192 ± 163	286 ± 143	446 ± 298	^*^8.6E-7
# COMT inhibitor	8 (33.3%)	20 (23.5%)	28 (50.0%)	^*^5.3E-3
# Anticholinergic agent	1 (4.2%)	9 (10.6%)	4 (7.1%)	0.64
# Dopamine agonist	11 (45.8%)	56 (65.9%)	39 (69.6%)	0.13
# MAO-B inhibitor	11 (45.8%)	17 (20.0%)	18 (32.1%)	^*^0.030
# Amantadine	1 (4.2%)	12 (14.1%)	16 (28.6%)	^*^0.016
# Smoking (never, past, present)	20 (83.3%), 2 (8.3%), 2 (8.3%)	69 (81.2%), 12 (14.1%), 4 (4.7%)	46 (82.1%), 9 (16.0%), 1 (1.8%)	0.62
# Coffee (none, 1 or 2/week, 3 ~ 5/week, 6 or 7/week)	1 (4.2%), 3 (12.5%), 3 (12.5%), 17 (70.8%)	16 (18.8%), 22 (25.9%), 12 (14.1%), 35 (41.2%)	14 (25%), 11 (19.6%), 8 (14.3%), 23 (41.1%)	0.15
Walking and balance (MDS-UPDRS 2.12)	0.708 ± 0.455	0.988 ± 0.728	1.66 ± 1.04	^*^5.9E-7
	(range 0–1)	(range 0–3)	(range 0–4)	
Freezing (MDS-UPDRS 2.13)	0.375 ± 0.563	0.600 ± 0.755	1.23 ± 1.07	^*^1.1E-5
	(range 0–2)	(range 0–2)	(range 0–4)	
Gait (MDS-UPDRS 3.10)	0.500 ± 0.500	0.976 ± 0.782	1.52 ± 0.886	^*^6.1E-7
	(range 0–1)	(range 0–3)	(range 0–4)	
Freezing of gait (MDS-UPDRS 3.11)	0 ± 0	0.165 ± 0.456	0.625 ± 0.836	^*^3.1E-6
	(range 0–0)	(range 0–2)	(range 0–4)	
Postural stability (MDS-UPDRS 3.12)	0.750 ± 0.968	0.506 ± 0.806	2.16 ± 1.03	^*^2.2E-16
	(range 0–3)	(range 0–3)	(range 0–4)	
Tremor (MDS-UPDRS 2.10)	0.542 ± 0.763	0.918 ± 0.785	1.00 ± 0.964	0.085
	(range 0–3)	(range 0–3)	(range 0–3)	
Postural tremor of the hands (MDS-UPDRS 3.15)	0.625 ± 0.633	0.894 ± 1.12	1.16 ± 1.08	0.098
	(range 0–2)	(range 0–6)	(range 0–4)	
Kinetic tremor of the hands (MDS-UPDRS 3.16)	0.833 ± 0.898	0.965 ± 1.30	1.39 ± 1.45	0.10
	(range 0–3)	(range 0–6)	(range 0–4)	
Rest tremor of the hands (MDS-UPDRS 3.17)	0.625 ± 0.949	0.482 ± 1.04	0.643 ± 1.33	0.68
	(range 0–3)	(range 0–6)	(range 0–5)	
Rest tremor of the legs (MDS-UPDRS 3.17)	0.417 ± 0.862	0.188 ± 0.642	0.35 ± 0.785	0.40
	(range 0–3)	(range 0–4)	(range 0–4)	
Rest tremor of the lip/jaw (MDS-UPDRS 3.17)	0 ± 0	0.0353 ± 0.323	0.0893 ± 0.391	0.47
	(range 0–0)	(range 0–3)	(range 0–2)	
Constancy of rest tremor (MDS-UPDRS 3.18)	0.708 ± 1.10	0.659 ± 1.18	0.571 ± 1.13	0.86
	(range 0–4)	(range 0–4)	(range 0–4)	
MMSE	29.3 ± 0.89	28.5 ± 2.3	27.6 ± 2.5	^*^6.6E-3
	(range 27–30)	(range 16–30)	(range 19–30)	

^a^Mean and SD are indicated when applicable.

^b^Either analysis of variance (ANOVA) or Fisher’s exact test is applied.

*P < 0.05.

### Construction of random forest models to predict whether HY stages are advanced in two years or not

**Fig. 1:**
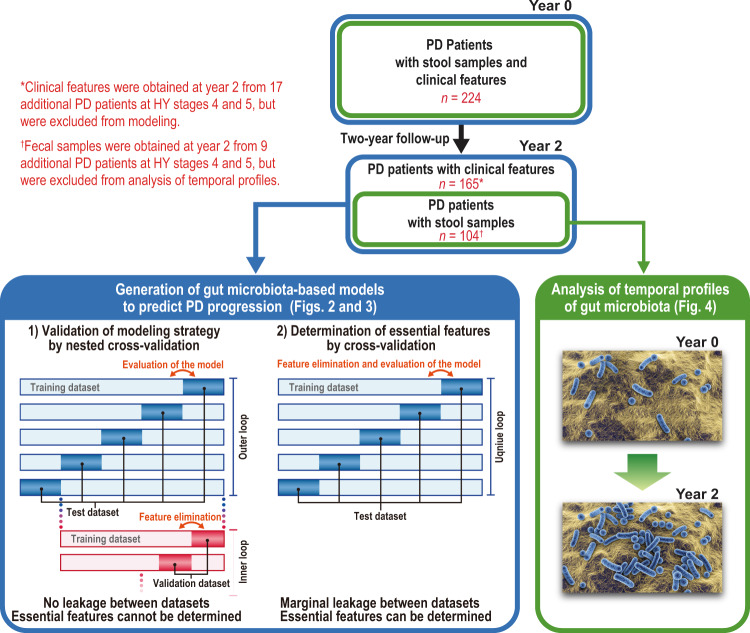
Illustration of workflows of this study. Fecal samples and clinical features were obtained at year 0 in 224 PD patients. At year 2, clinical features were evaluated in 165 PD patients, and fecal samples were obtained in 104 PD patients. Although clinical features were evaluated at year 2 in 17 additional PD patients (a total of 182 patients), they were excluded from making prediction models (see Materials and Methods). Similarly, fecal samples were obtained at year 2 in nine additional PD patients (a total of 113 patients), but they were excluded from analysis of temporal profiles of gut microbiota. Bacterial and clinical features at year 0, as well as clinical features at year 2, in 165 PD patients were used for Figs. 2 and 3. Gut microbiota at years 0 and 2 in 104 PD patients were used for Fig. 4. Construction of prediction models was constituted of two steps: (1) nested cross-validation has no leakage between the training and test datasets, and is for evaluation of the modeling strategy, and (2) cross-validation has marginal leakage between the training and test datasets, and is for determination of essential features to predict the progression of PD. Recursive feature elimination (FRE) was employed in both steps. AUROCs were calculated in both steps, but AUROC of the nested cross-validation should be dependable because of lack of potential leakage.

We divided PD patients at HY stages 1–3 (*n* = 165), 1 (*n* = 24), 2 (*n* = 85), and 3 (*n* = 56) at year 0 into the deteriorated and stable groups. The deteriorated group had an increased HY stage, whereas the stable group had an unchanged or decreased HY stage. We made random forest models to differentiate the deteriorated and stable groups for HY stages 1–3, 1, 2, and 3 at year 0 using bacterial features, and compared the models with those using clinical features. Generations of random forest models by nested cross-validation and cross-validation are indicated in detail in Methods, and are illustrated in Fig. 1. We first examined the validity of our modeling strategy by nested cross-validation with recursive feature elimination (RFE), which should have no leakage (type 1 circularity^49^) between the training and test datasets.

**Fig. 2:**
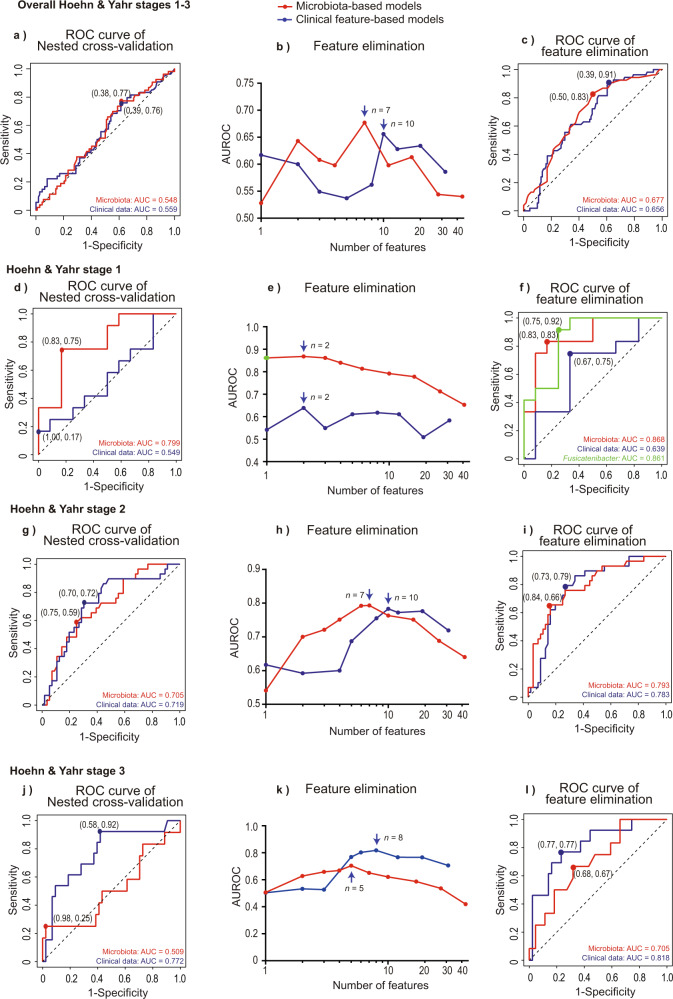
Validation of modeling strategy by nested cross-validation and determination of essential features by cross-validation. **a, d, g,** and **j** ROC curves of nested cross-validation of random forest models for HY stages 1–3 (**a**), 1 (**d**), 2 (**g**), and 3 (**j**) at year 0. Red and blue solid lines represent models constructed by bacterial and clinical features, respectively. The optimal point by Youden index is indicated by a dot with the specificity and sensitivity in parentheses. **b, e, h,** and **k** AUROCs by leave-one-out cross-validation of random forest models for HY stages 1–3 (**b**), 1 (**e**), 2 (**h**), and 3 (**k**) at year 0, while features were recursively eliminated. An arrow points to the maximum AUROC with the number of features. **c, f, i,** and **l** ROC curves of leave-one-out cross-validation of random forest models at the maximum AUROC for HY stages 1–3 (**c**), 1 (**f**), 2 (**i**), and 3 (**l**) at year 0. The optimal point by Youden index is indicated by a dot with the specificity and sensitivity in parentheses. Green ROC curve in **f** represents a model predicted by *Fusicatenibacter* alone, and its AUROC is plotted in green in **e**.

The nested cross-validation for HY stages 1–3 at year 0 yielded AUROCs of 0.548 (95% confidence interval: 0.456–0.641) in microbiota-based models (red line in Fig. 2a) and 0.559 (0.464–0.654) in clinical feature-based models (blue line in Fig. 2a). Thus, both bacterial and clinical features failed to make decent models. We thus constructed models for each HY stage.

For HY stage 1 at year 0, the nested cross-validation yielded AUROCs of 0.799 (95% confidence interval: 0.615–0.982) in microbiota-based models (red line in Fig. 2d) and 0.549 (0.307–0.791) in clinical feature-based models (blue line in Fig. 2d). In the nested cross-validation, the microbiota-based model yielded 75% sensitivity and 83% specificity, whereas the clinical feature-based model failed to make a decent model (Fig. 2d). Seven statistical measures including sensitivities and specificities to indicate the performance of each model were all better with the microbiota-based models than with the clinical feature-based models (Table 2). As the nested cross-validation did not provide us with essential features that determined the progression of PD, we made models by recursively eliminating features and evaluated them by cross-validation (Fig. 1). Recursive feature elimination with cross-validation showed that maximum AUROCs were 0.868 (0.719–1.000) at two genera (*Fusicatenibacter* and *Faecalibacterium*) in microbiota-based models (red line in Fig. 2e, f) and 0.639 (0.402–0.876) at two features (BMI and age) in clinical feature-based models (blue line in Fig. 2e, f). AUROC was as high as 0.861 (0.707–1.000) even when a model was generated using genus *Fusicatenibacter* alone (green dot in Fig. 2e and green line in Fig. 2f). The microbiota-based model with *Fusicatenibacter* and *Faecalibacterium* yielded 83% sensitivity and 83% specificity, and *Fusicatenibacter*-based model yielded 92% sensitivity and 75% specificity (Fig. 2f). In contrast, the clinical feature-based model with BMI and age yielded 75% sensitivity and 67% specificity (Fig. 2f).

**Table 2:** Area under the ROC curve (AUROC) and seven statistical measures of random forest models for Hoehn & Yahr (HY) stages 1–3, 1, 2, and 3.

			AUROC	95% Confidence Interval	Accuracy	Positive Predictive Value (PPV)	Sensitivity	Specificity	F1 score	Negative Predictive Value (NPV)	MCC
Nested cross-validation	Bacterial feature-based models	HY stages 1–3	0.548	0.456–0.641	–	–	–	–	–	–	–
		HY stage 1	0.799	0.615–0.982	79.2%	81.8%	75.0%	83.3%	78.3%	76.9%	0.585
		HY stage 2	0.705	0.592–0.818	69.4%	54.8%	58.6%	75.0%	56.7%	77.8%	0.331
		HY stage 3	0.509	0.301–0.719	–	–	–	–	–	–	–
	Clinical feature-based model	HY stages 1–3	0.559	0.464–0.654	–	–	–	–	–	–	–
		HY stage 1	0.549	0.307–0.791	–	–	–	–	–	–	–
		HY stage 2	0.719	0.602–0.835	70.6%	55.3%	72.4%	69.6%	62.7%	83.0%	0.401
		HY stage 3	0.772	0.619–0.925	66.1%	40.0%	92.3%	58.1%	55.8%	96.2%	0.427
Cross-validation	Bacterial feature-based models	HY stages 1–3	0.677	0.592–0.762	60.6%	44.0%	83.0%	50.0%	57.5%	86.2%	0.316
		HY stage 1	0.868	0.719–1.000	83.3%	83.3%	83.3%	83.3%	83.3%	83.3%	0.667
		HY stage 2	0.793	0.692–0.895	77.7%	67.9%	65.5%	83.9%	66.7%	82.5%	0.499
		HY stage 3	0.705	0.544–0.865	67.9%	36.4%	66.7%	68.2%	47.0%	88.2%	0.293
	*Fusicatenibacter*	HY stage 1	0.861	0.707–1.000	83.3%	78.6%	91.7%	75.0%	84.6%	90.0%	0.676
	Clinical feature-based model	HY stages 1–3	0.656	0.571–0.741	55.8%	41.9%	90.7%	38.7%	57.3%	89.6%	0.305
		HY stage 1	0.639	0.402–0.876	70.8%	69.2%	75.0%	66.7%	72.0%	72.7%	0.418
		HY stage 2	0.783	0.682–0.883	68.7%	60.5%	79.3%	73.2%	68.7%	87.2%	0.501
		HY stage 3	0.818	0.682–0.953	76.8%	50.0%	76.9%	76.7%	60.6%	91.7%	0.473

The number of PD patients at HY stages 1, 2, and 3 at year 0 were 24, 85, and 56, respectively.

The seven following statistical measures were calculated when AUROC was >0.6.

For HY stage 2 at year 0, the nested cross-validation yielded AUROCs of 0.705 (0.592–0.818) in microbiota-based models (red line in Fig. 2g) and 0.719 (0.602–0.835) in clinical feature-based models (blue line in Fig. 2g). Seven statistical measures to indicate the performance of each model were slightly better with the clinical feature-based models than with the microbiota-based models except for specificity (Table 2). Recursive feature elimination with cross-validation showed that maximum AUROCs were 0.793 (0.692–0.895) at seven genera (*Lactobacillus, Blautia*, *Fusicatenibacter*, *Anaerostipes*, *Ruminococcus gnavus group*, *Akkermansia*, *Bifidobacterium*) in microbiota-based models (red line in Fig. 2h, i) and 0.783 (0.682–0.883) at ten features in clinical feature-based models (blue line in Fig. 2h, i).

For HY stage 3 at year 0, the nested cross-validation yielded AUROCs of as low as 0.509 (0.301–0.719) in microbiota-based models (red line in Fig. 2j) and 0.772 (0.619–0.925) in clinical feature-based models (blue line in Fig. 2j). Thus, microbiota-based models were dependable for the early stage of PD, but clinical feature-based models became reliable with the advancement of PD.

We also made random forest models using both bacterial and clinical features to examine whether some clinical features were as essential as bacterial features for HY stage 1 (Supplementary Fig. S1, Supplementary Table S3). Step-wise feature elimination for HY stage 1 revealed that the combined feature-based models and the gut microbiota-based models (black solid line and red dotted line in Supplementary Fig. S1b, respectively) became identical, when the number of features became six or less. Thus, all clinical features were eliminated in the combined feature-based models, when the number of features became six or less. This indicates that none of the 31 clinical features were as predictive as the remaining six bacterial features. Two bacterial features made the maximum AUROC (an arrow in Supplementary Fig. S1b), and are indicated in Supplementary Table S3.

In contrast, clinical features constituted two out of nine essential features for HY stage 2, and three out of six essential features for HY stage 3 (Supplementary Table S3). This indicates that some clinical and some bacterial features were similarly essential to predict the progression of PD for HY stages 2 and 3. However, nested cross-validation showed that the combined models were not as good as either microbiota-based models or clinical feature-based models for each HY stage, which was likely due to the inclusion of a large number of non-informative features. In contrast to nested cross-validation (Supplementary Fig. S1d, g), cross-validation showed that the combined models outperformed both microbiota-based models and clinical feature-based models (Supplementary Fig. S1e, f, h, i) for HY stages 2 and 3, which indicates the requirement of nested cross-validation to examine the feasibility of modeling strategies.

We next asked whether gut microbiota is able to predict changes of MDS-UPDRS III, representing objective motor symptoms, in two years. As gut microbiota was able to predict the progression of PD at the early stage of PD, we divided PD patients in half using MDS-UPDRS III to make cohorts of the early and advanced stages of PD patients. We then sorted the rates of change of MDS-UPDRS III in two years in ascending order. The top and bottom halves of patients constituted the stable and deteriorated groups, respectively. Nested cross-validation of microbiota-based models for the early and advanced PD patients yielded AUROCs of 0.728 (95% confidence interval: 0.601–0.854) and 0.586 (95% confidence interval: 0.449–0.723), respectively. Cross-validation of microbiota-based models identified four essential genera. Genera *Faecalibacterium*, *Dorea*, *Ruminococcus gnavus group* were decreased, while genus *Bacteroides* was increased, in the deteriorated group. In HY-based models, genera *Faecalibacteirum* and *Ruminococcus gnavus group* constituted essential features in HY stage 1 and 2, respectively. Genera *Faecalibacterium* and *Dorea* are SCFA-producing bacteria. Identification of the early stage of PD and evaluation of the disease progression both by MDS-UPDRS III similarly showed that gut microbiota predicted the progression of PD in two years for the early stage of PD.

As we evaluated all PD patients in outpatient clinics, MDS-UPDRS III scores were obtained in the “on” state. We estimated MDS-UPDRS III scores in the “off” state by adding 7.3, 8.5 and 6.1 for patients taking levodopa only, levodopa and any other medications, and dopamine agonists without levodopa according to a report by Bordelon and colleagues^50^. Nested cross validation with the adjusted MDS-UPDRS III scores in the “off” state yielded AUROC of 0.717 (0.550–0.883) and 0.571 (0.444–0.697) for the early stage and the advanced stages of PD, respectively, which were similar to those obtained with the “on” state. We examined whether combinations of medications, as well as levodopa-equivalent daily doses (LEDDs), were different between the stable and deteriorated groups at years 0 and 2. We found that combinations of medications were not statistically different between the stable and deteriorated groups at years 0 and 2 (Supplementary Table S4). Although the rate of change of LEDDs in two years was marginally higher in the stable group than the deteriorated group, no statistical significance was observed (Supplementary Fig. S2).

### Differences of taxonomic relative abundances between the deteriorated and stable groups

**Fig. 3:**
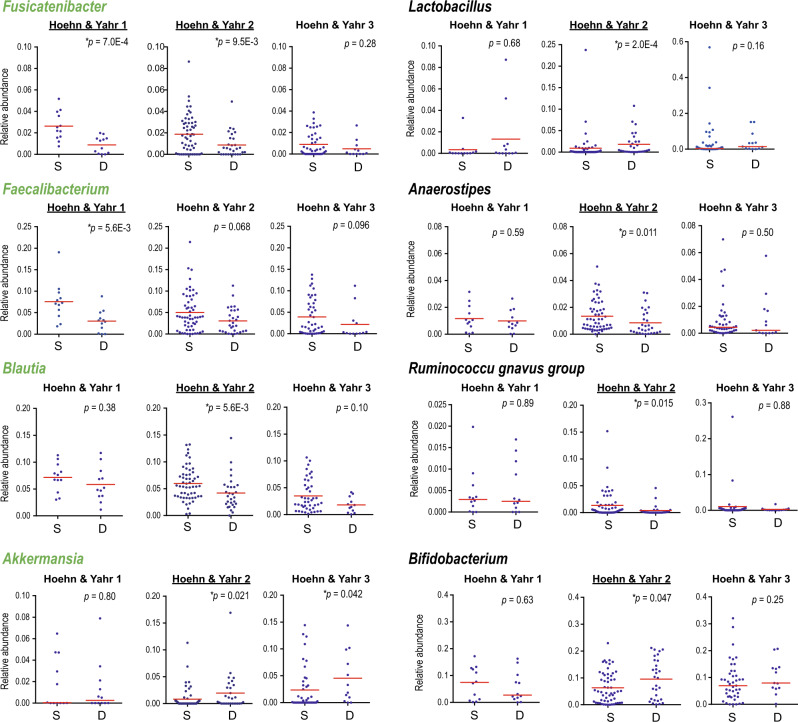
Relative abundances of essential genera at year 0, which made the maximum AUROCs for HY stages 1 and 2 in Fig. 2eh, in the stable (S) and deteriorated (D) groups. HY stages for which a specific genus was essential in modeling are underlined. Genera that were significantly changed in our dataset in our previous analysis^38^ are indicated in green letters. Note that vertical scales are different for different HY stages in *Akkermansia*, *Lactobacillus*, and *Ruminococcus* *gnavus* *group*. Means are indicated by red bars. *P*-values were calculated by the Wilcoxon rank sum test. ^*^P < 0.05.

We collated the feature importance and *p*-value of bacterial and clinical features that attained a maximum AUROC for HY stages 1, 2, and 3 in Supplementary Tables S5 and S6, respectively. Again, microbiota-based models for HY stages 1 and 2, but not HY stage 3, were dependable. Two and seven essential genera made dependable models for HY stages 1 and 2, respectively, while genus *Fusicatenibacter* was shared between HY stages 1 and 2. To ask why essential genera were different between HY stages 1 and 2, we plotted relative abundances of the eight genera in the deteriorated and stable groups for each of HY stages 1, 2, and 3 (Fig. 3). We found that these genera were changed in the same direction in the stable and deteriorated groups for HY stages 1, 2, and 3, except for genus *Bifidobacterium* in HY stage 1. The same genera were likely to have the same effects on the progression of PD independent of HY stages, but the effect size of each genus was different from HY stage to HY stage. It was also interesting to note that *Fusicatenibacter*, *Faecalibacterium*, *Blautia*, and *Akkermansia* (green letters in Fig. 3) were significantly different between controls and PD in our data in our previous analysis^38^.

### Change of relative abundances of four genera in two years

**Fig. 4:**
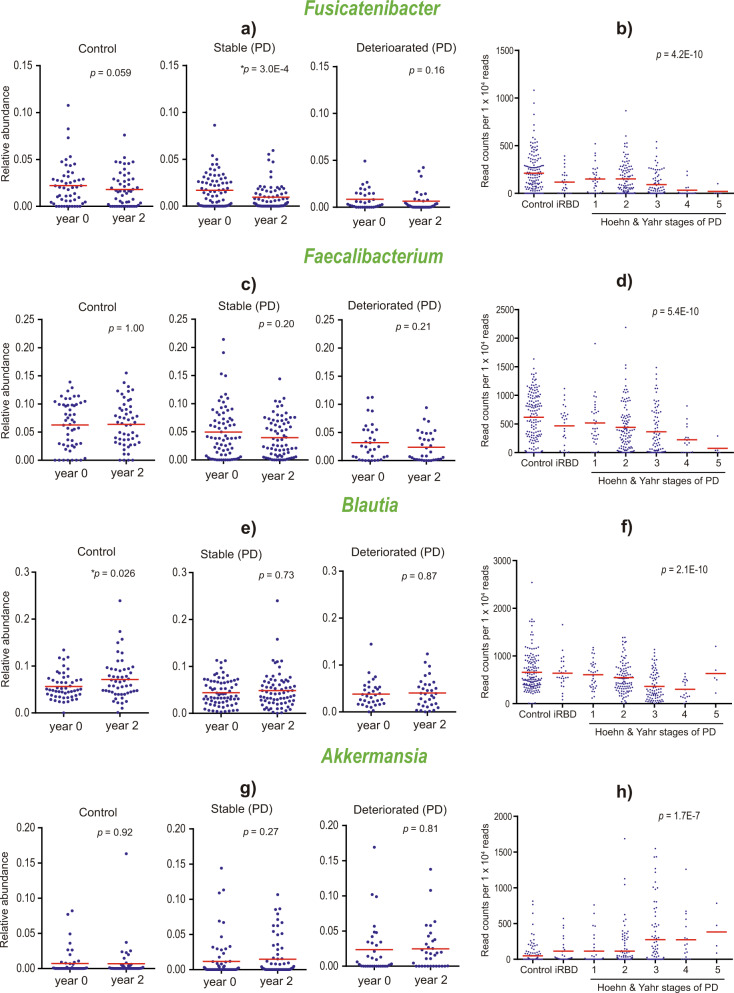
Changes of four essential genera in two years and in the course of disease progression. Relative abundances of genera *Fusicatenibacter*, *Faecalibacterium*, *Blautia*, and *Akkermansia* at years 0 and 2 in the control, stable, and deteriorated groups of combined HY stages 1–3 (**a, c, e,** and **g**), as well as at year 0 in controls, iRBD, and HY stages 1–5 (**b, d, f,** and **h**). The four genera constituted features to make the highest AUROC models (Fig. 2 and Supplementary Table S5) and were significantly changed in PD in our dataset in our previous analysis^38^. Means are indicated by red bars. **a, c, e,** and **g** *P*-values were calculated by the Wilcoxon signed-rank test. ^*^P < 0.05. Means are indicated by red bars. **b, d, f,** and **h** *P*-values are calculated by Jonckheere-Terpstra trend test to indicate whether the relative abundances increase or decrease monotonically. Plots of genera *Faecalibacterium* (**d**) and *Akkermansia* (**h**) with the progression of α-synucleinopathy were previously reported with fewer numbers of samples^42^.

Plots of the four genera, *Fusicatenibacter*, *Faecalibacterium*, *Blautia*, and *Akkermansia*, at years 0 and 2 in the deteriorated group showed that all the four genera remained unchanged in two years (Fig. 4a, c, e, g). Additionally, plots of these four genera in the course of progression of α-synucleinopathy showed that *Akkermansia* becomes higher with disease progression, whereas *Fusicatenibacter*, *Faecalibacterium* and *Blautia* become lower with disease progression, with statistically significant monotonous trends for all the four genera (Fig. 4b, d, f, h). No change of the four genera even in the deteriorated group and the change of the four genera with disease progression suggest that patients harboring dysbiosis of the four genera progress more rapidly than those without dysbiosis.

## Discussion

We examined whether gut microbiota was able to predict the progression of PD in two years, and asked whether gut microbiota predicted the progression of PD more precisely than clinical features. We first scrutinized whether our modeling approach was appropriate or not by nested cross-validation, in which there should be no leakage between the training and test datasets (Fig. 1). Nested cross-validation showed that bacterial features predicted the progression of PD with AUROC = 0.799 for HY stage 1, and the efficiency decreased for HY stages 2 and 3 (Fig. 2 and Table 2). Bacterial features similarly predicted the progression of PD evaluated by MDS-UPDRS III with AUROC = 0.728 for the early stage of PD. Similarly, bacterial features predicted the progression of PD evaluated by MDS-UPDRS III in the estimated “off” state with AUROC = 0.717 for the early stage of PD. However, lack of the MDS-UPDRS III in the “off” state in our patients was a limitation of our analysis. In contrast to gut microbiota-based models, nested cross-validation showed that clinical features predicted the progression of PD with AUROC = 0.772 for HY stage 3, and the efficiency decreased for HY stages 2 and 1 (Fig. 2 and Table 2). Thus, bacterial and clinical features predicted the progression of PD at the early and medium stages of PD, respectively.

Two and seven genera were essential to predict the disease progression for HY stages 1 and 2, respectively, and only genus *Fusicatenibacter* was shared between HY stages 1 and 2 (Fig. 3 and Supplementary Table S5). Plots of the relative abundances of the eight genera showed that seven out of the eight genera had the same effects on the progression of PD for HY stages 1, 2, and 3 (Fig. 3). The difference in the effect sizes of each genus for different HY stages was likely to have given rise to different essential genera for each HY stage.

Clinical features were better than bacterial features in predicting the progression of PD for HY stage 3, which was likely because at HY stage 1 all PD patients exhibited minimal and similar clinical phenotypes, and clinical features were not informative enough to predict the prognosis. In contrast to HY stage 1, PD patients at HY stage 3 exhibited widely variable phenotypes from infrequent episodes of toppling down to marked difficulty in walking. Among a total of 12 essential clinical features to predict the prognosis of PD for HY stages 1, 2, and 3 (Supplementary Table S6), six features (age, MMSE, tremor, postural instability, walking and balance, and gait) were previously reported to be associated with the progression and the mortality rate of PD in two original articles^18,21^ and a review article summarizing 27 original articles^17^. Two features (disease duration and levodopa dosage) were also predictive of the progression and the mortality rate of PD in some but not in all reports^17,18,21^. The remaining four features (BMI, COMT inhibitor, kinetic tremor of hands, and stool frequency) have not been analyzed in association with the progression and the mortality rate of PD to the best of our knowledge. Both previous studies^17–21^ and our current study point to the notion that tremor-dominant type of PD progresses more slowly than postural instability/gait-dominant type of PD.

A machine-learning model was recently reported to predict the prognosis of PD using clinical features, which was aimed at predicting the progression of total MDS-UPDRS in two or four years^22^. AUROC of their model was 0.70^22^. The important features were autonomic dysfunction, mood impairment, anxiety, iRBD, cognitive decline, and memory impairment^22^. MMSE in our model for HY stage 2, and cognitive decline and memory impairment in a model for total MDS-UPDRS^22^, were the only important features shared between the two models. Lack of shared features may be accounted for by the differences in the number of features: 601 features in their model vs 31 features in our model.

After assuring the validity of our modeling strategy by nested cross-validation, we determined essential bacterial features by cross-validation with RFE (Fig. 1). For HY stage 1, the best model to predict the progression of PD was constructed by two genera (*Fusicatenibacter* and *Faecalibacterium*) (Supplementary Table S5). Similarly, for HY stage 2, the best model was constructed by seven genera including *Fusicatenibacter* and *Blautia* (Supplementary Table S5). These three genera (*Fusicatenibacter*, *Faecalibacterium*, and *Blautia*) are SCFA-producing bacteria. *Faecalibacterium* was decreased in PD across countries in meta-analyses by us^38^ and others^51^. Similarly, *Fusicatenibacter* and *Blautia* were decreased in PD except for Germany in meta-analyzes by us^38^ and others^51^. Reduced abundances of these genera were thus hallmarks of rapid progression in the early stage of PD, as well as hallmarks of PD. As stated in the introduction, decreased SCFAs are potentially associated with abnormal activation of neuroinflammations in the brain^47,48^. In addition, increased genus *Akkermansia* also predicted the progression of PD for HY stage 2 in our model (Supplementary Table S5). In contrast, *Akkermansia* is protective against ALS^52^ and epilepsy^53^ in mouse models, as well as diabetes mellitus^54–56^ and obesity^57–60^ in humans. In a mouse model of ALS, nicotinamide produced by *Akkermansia* improves motor symptoms^52^. In a mouse model of epilepsy, ketogenic-diet increases *Akkermansia*, which inhibits seizure by decreasing gamma-glutamylated amino acids in the colon lumen^53^. As epidemiological studies indicate that diabetes mellitus increases a risk of PD 1.85-folds^61^, *Akkermansia* should decrease a risk of PD by normalizing glucose metabolisms^54–56^. We, however, showed that *Akkermansia* was rather associated with the development and progression of PD. *Akkermansia*-mediated improvement in glucose metabolisms may have no effect on the prevention of the development of PD by unknown mechanisms. *Akkermansia* thickens the mucin layer of mice when fed with a high fat diet^45^. On the other hand, *Akkermansia* degrades the mucin layer in mice when fed with fiber-free diet^44^. Similarly, *Akkermansia* induces intestinal inflammation and increases intestinal permeability by possibly generating hydrogen sulfide^62^. The intestinal environment of PD patients is likely to be similar to the latter situation since expression of the tight junction protein, occludin, is decreased in PD^63^, and intestinal permeability is increased in PD^23,46^. The association of pesticides and herbicides with PD has been repeatedly reported in 440 original epidemiological studies and 69 review articles^64^. The increased intestinal permeability might have led to exposure of the intestinal nerve plexus to pesticides/herbicides and other toxins. Alternatively, increased intestinal permeability may enhance the intestinal oxidative stress, as observed in increased intestinal staining for nitrotyrosine in PD patients^46^, which may potentiate the formation of α-synuclein fibrils. Increased *Akkermansia* is thus likely to have substantial effects on the development and progression of PD, but about 42% of PD patients had no intestinal *Akkermansia* in our cohort^38^. PD in patients without *Akkermansia* is likely to be mediated by SCFA-producing or other unrecognized bacteria, or not by dysbiosis of gut microbiota.

Although we obtained stool samples in 50.4% of PD patients at year 2, we unexpectedly observed that relative abundances of four genera (*Fusicatenibacter*, *Faecalibacterium, Blautia*, and *Akkermansia*) remained unchanged in two years even in the deteriorated group (Fig. 4a, c, e, g). Nevertheless, we observed that genus *Akkermansia* was increased with the progression of α-synucleinopathy, whereas genera *Fusicatenibacter, Faecalibacterium*, and *Blautia* were decreased with the progression (Fig. 4b, d, f, h). Thus, increased *Akkermansia*, and decreased *Fusicatenibacter, Faecalibacterium*, and *Blautia* were not due to the progression of PD, but were likely to have driven the progression of PD. In other words, PD patients with these taxonomic changes were likely to be destined to progress rapidly. These observations are also in accordance with the assumption that intestinal dysbiosis of these genera determines the progression of α-synucleinopathy. We thus may be able to retard the progression of PD in the early stages by therapeutic intervention with pre-, pro-, and post-biotics to normalize gut dysbiosis or to compensate for defective gut metabolisms.

## Methods

### Patients

All studies were approved by the Ethical Review Committees of the Nagoya University Graduate School of Medicine (approval #2016-0151), Iwate Medical University (H28-123), Okayama Kyokuto Hospital (approval #kyoIR-2016002), and Fukuoka University School of Medicine (approval #2016M027). We obtained written informed consent from all recruited individuals.

Out of 251 potentially eligible patients at year 0 (November 2016–May 2019), 27 patients did not participate in this study. We thus obtained fecal samples and clinical features in 224 PD patients at year 0 (November 2016–May 2019), and followed them for two years. PD patients were diagnosed based on the Movement Disorder Society’s (MDS) PD criteria^65^. Our cohort did not include PD patients with other chronic diseases including diabetes mellitus, heart failure, liver cirrhosis, malignancy, hematological diseases, and autoimmune diseases. Similarly, our cohort did not include PD patients who claimed to have taken antibiotics in the past one month. At year 2 (November 2018–May 2021), fecal samples were obtained from 113 PD patients, and clinical features were obtained from 182 PD patients. We obtained clinical features at year 2 in 182 out of 224 PD patients (81.3%). We similarly obtained fecal samples at year 2 in 113 PD patients (50.4%). Loss of a substantial number of patients in 2 years was partly because participants complained that it was too much burden to take and send fecal samples to us. Additionally, some participants moved out to other hospitals. We also included 137 healthy controls whose fecal samples were available at year 0. Among the controls, 112 subjects were healthy cohabitants of PD patients. At year 2, fecal samples were obtained from 52 controls.

### DNA isolation and 16S rRNA V3–V4 gene amplicon sequencing

The samples were transported from the participant’s home to Nagoya University below 4 ˚C, freeze-dried^66^, and subjected to DNA isolation and sequencing of the 16S rRNA V3–V4 region using a pair of primers (341F, 5'-CCTACGGGNGGCWGCAG-3' and 805R, 5'-GACTACHVGGGTATCTAATCC-3')^38,42^. Paired-end sequencing of 300-nucleotide fragments was performed using the MiSeq reagent kit V3 on a MiSeq System (Illumina). The 16S rRNA gene amplicon sequencing data were analyzed by QIIME2^67^ with DADA2 using the SILVA taxonomy database release 138^68,69^.

### Deteriorated and stable groups for combined HY stages 1–3 and each of HY stages 1, 2, and 3

For HY stages 1–3 (165 patients), 1 (24 patients), 2 (85 patients), and 3 (56 patients), we divided PD patients into the deteriorated and stable groups. The deteriorated group was comprised of PD patients with an advanced HY stage at year 2 compared to year 0. In contrast, in the stable group, the HY stage remained unchanged or was decreased at year 2. We excluded 14 patients at HY stage 4 at year 0, because only two of them were advanced to HY stage 5 in two years. The unbalanced dataset should give rise to a biased model that would be in favor of predicting no progression. We also excluded three patients with HY stage 5 at year 0, because this was the final stage of PD.

We obtained 113 pairs of stool samples at years 0 and 2. To analyze taxonomic changes in two years, we excluded nine pairs of stool samples with HY stages 4 and 5 at year 0. A total of 104 pairs of stool samples were thus used to analyze taxonomic changes in two years (Fig. 1).

### Bacterial and clinical features for random forest modeling

We filtered intestinal genera under the following conditions. For each dataset, we selected genera with >0.5% relative abundance on average. The numbers of genera that satisfied this criterion were 44, 41, 43, and 42 for HY stages 1–3, 1, 2, and 3, respectively. These genera were used as features to predict whether HY stages were advanced or not in two years for each dataset. We similarly limited the number of clinical and demographic features to 31 to prevent overfitting of our models and also to match the number of bacterial features. The clinical and demographic features included age, sex, body mass index (BMI), disease duration, stool frequency per week, and HY stage at year 0. The clinical features also included the use of proton pump inhibitor, H_2_ blocker, antihyperlipidemic drug, angiotensin II receptor blocker, calcium channel blocker, COMT inhibitor, anticholinergic agent, dopamine agonist, MAO-B inhibitor, and amantadine, as well as levodopa/carbidopa dosage. We also used MDS-UPDRS to differentiate dominance in tremor and postural instability with gait difficulty^70^. We assessed MDS-UPDRS in PD patients with medication ON state. The extracted MDS-UPDRS features included tremor (MDS-UPDRS 2.10), walking and balance (MDS-UPDRS 2.12), freezing (MDS-UPDRS 2.13), gait (MDS-UPDRS 3.10), freezing of gait (MDS-UPDRS 3.11), postural stability (MDS-UPDRS 3.12), postural tremor of the hands (MDS-UPDRS 3.15), kinetic tremor of the hands (MDS-UPDRS 3.16), rest tremor of the hands (MDS-UPDRS 3.17), rest tremor of the legs (MDS-UPDRS 3.17), rest tremor of the lip/jaw (MDS-UPDRS 3.17), and constancy of rest tremor (MDS-UPDRS 3.18). We also included Mini-Mental State Examination (MMSE), coffee intake, and smoking.

### Construction of random forest models to predict whether HY stages are advanced in two years or not

We constructed random forest models with sklearn.ensemble.RandomForestRegressor function on Python 3.8.2 to differentiate the deteriorated and stable groups for HY stages 1–3, 1, 2, and 3 at year 0 using bacterial and clinical features. We followed the AUC-RF method to determine the bacterial and clinical features^71^, which was previously adopted to make random forest models using bacterial features to differentiate adenoma and colon cancer^72^. The outline of this analysis was illustrated in Fig. 1. We first examined the performance of our modeling strategy by nested cross-validation with recursive feature elimination using sklearn.feature_selection.RFECV function on Python 3.8.2. In the nested cross-validation, the outer loop was comprised of leave-one-out cross validation (LOOCV), whereas the inner loop was comprised of 10-to-20-fold cross validation depending on the number of samples. In the inner loop, features were recursively eliminated one by one to obtain the best combination of features that gave rise to the highest AUROC. The best model in the inner loop was generated, and was applied to predict the prognosis of a patient that was left out by LOOCV. The nested cross-validation should have no leakage between the training and test datasets (type 1 circularity^49^), but could not provide us with bacterial and clinical features to make clinically applicable models. We thus determined essential bacterial and clinical features by recursive feature elimination using the sklearn.feature_selection.RFE function on Python 3.8.2. For the determined essential genera, we compared the relative abundances in the stable and deteriorated groups at year 0 by the Wilcoxon rank sum test. Python code to perform nested cross-validation and cross-validation are available upon request.

### Seven statistical measures to represent the model performance

We evaluated the performance of random forest models by the following seven statistical measures. TP, FP, FN, and TN indicate true positive, false positive, false negative, and true negative, respectively.

1 $${{{\mathrm{Accuracy}}}} = \frac{{{{{\mathrm{TP}}}} + {{{\mathrm{TN}}}}}}{{{{{\mathrm{TP}}}} + {{{\mathrm{FP}}}} + {{{\mathrm{TN}}}} + {{{\mathrm{FN}}}}}}$$

Rate to predict true positives and true negatives in the whole dataset

2 $${{{\mathrm{Precision}}}}/{{{\mathrm{Positive}}}}\,{{{\mathrm{Prediciton}}}}\,{{{\mathrm{Value}}}}\,\left( {{{{\mathrm{PPV}}}}} \right) = \frac{{{{{\mathrm{TP}}}}}}{{{{{\mathrm{TP}}}} + {{{\mathrm{FP}}}}}}$$

Rate of true positives in predicted positives

3 $${{{\mathrm{Recall}}}}/{{{\mathrm{Sensitivity}}}} = \frac{{{{{\mathrm{TP}}}}}}{{{{{\mathrm{TP}}}} + {{{\mathrm{FN}}}}}}$$

Rate of true positives in actual positives

4 $${{{\mathrm{Specificity}}}} = \frac{{{{{\mathrm{TN}}}}}}{{{{{\mathrm{FP}}}} + {{{\mathrm{TN}}}}}}$$

Rate of true negatives in actual negatives

5 $${{{\mathrm{F}}}}1\,{{{\mathrm{score}}}} = 2\frac{{{{{\mathrm{Precision}}}} \times {{{\mathrm{Recall}}}}}}{{{{{\mathrm{Precision}}}} + {{{\mathrm{Recall}}}}}}$$

Harmonic mean of precision and recall. Higher precision and higher recall increase F1 score, but discrepancy between precision and recall lowers F1 score.

6 $${{{\mathrm{Negative}}}}\,{{{\mathrm{Predictive}}}}\,{{{\mathrm{Value}}}}\,({{{\mathrm{NPV}}}}) = \frac{{{{{\mathrm{TN}}}}}}{{{{{\mathrm{TN}}}} + {{{\mathrm{FN}}}}}}$$

Rate of true negatives in predicted negatives

7 $$\begin{array}{l}{{{\mathrm{Matthews}}}}\,{{{\mathrm{Correlation}}}}\,{{{\mathrm{Coefficient}}}}\,({{{\mathrm{MCC}}}})\\\,\, = \frac{{{{{\mathrm{TP}}}} \times {{{\mathrm{TN}}}}--{{{\mathrm{FP}}}} \times {{{\mathrm{FN}}}}}}{{\sqrt {({{{\mathrm{TP}}}} + {{{\mathrm{FP}}}})({{{\mathrm{TP}}}} + {{{\mathrm{FN}}}})({{{\mathrm{TN}}}} + {{{\mathrm{FP}}}})({{{\mathrm{TN}}}} + {{{\mathrm{FN}}}})} }}\end{array}$$

A correlation coefficient between the actual and predicted binary conditions while the numbers of each condition are balanced. Unlike the other parameters, MCC balances the ratio between actual positives and actual negatives.

### Statistical analysis

Relative abundances of intestinal bacteria were analyzed by the Wilcoxon signed-rank test for matched pairs with the wilcoxon functionality of scipy.stat, and by the Wilcoxon rank sum test for unmatched pairs with the mannwhitneyu functionality of scipy.stat, both on Python 3.8.2. Jonckheere-Terpstra trend test to examine whether intestinal bacteria increased or decreased monotonically was performed with jonckheere.test of library PMCMR on R version 4.1.0. The area under the receiver operating characteristic curve (AUROC) was calculated with the roc_curve functionality of sklearn.metrics on Python 3.8.2. *P*-values < 0.05 were considered to be significantly different.

## Supplementary information

SUPPLEMENTAL MATERIAL

## Data Availability

FASTQ files of our dataset are available at the DNA Data Bank of Japan (DDBJ) under the accession numbers of “DRA009229” and “DRA012438” for year 0, and “DRA012445” for year 2.
