# Supplementary material for: Short chain fatty acids-producing and mucin-degrading intestinal bacteria predict the progression of early Parkinson’s disease
Source: NPJ Parkinsons Dis. 2022 Jun 1;8:65. doi: 10.1038/s41531-022-00328-5 (PMC9160257; doi:10.1038/s41531-022-00328-5)
Supplement: Supplementary file 1 — SUPPLEMENTAL MATERIAL [file 41531_2022_328_MOESM1_ESM.pdf]

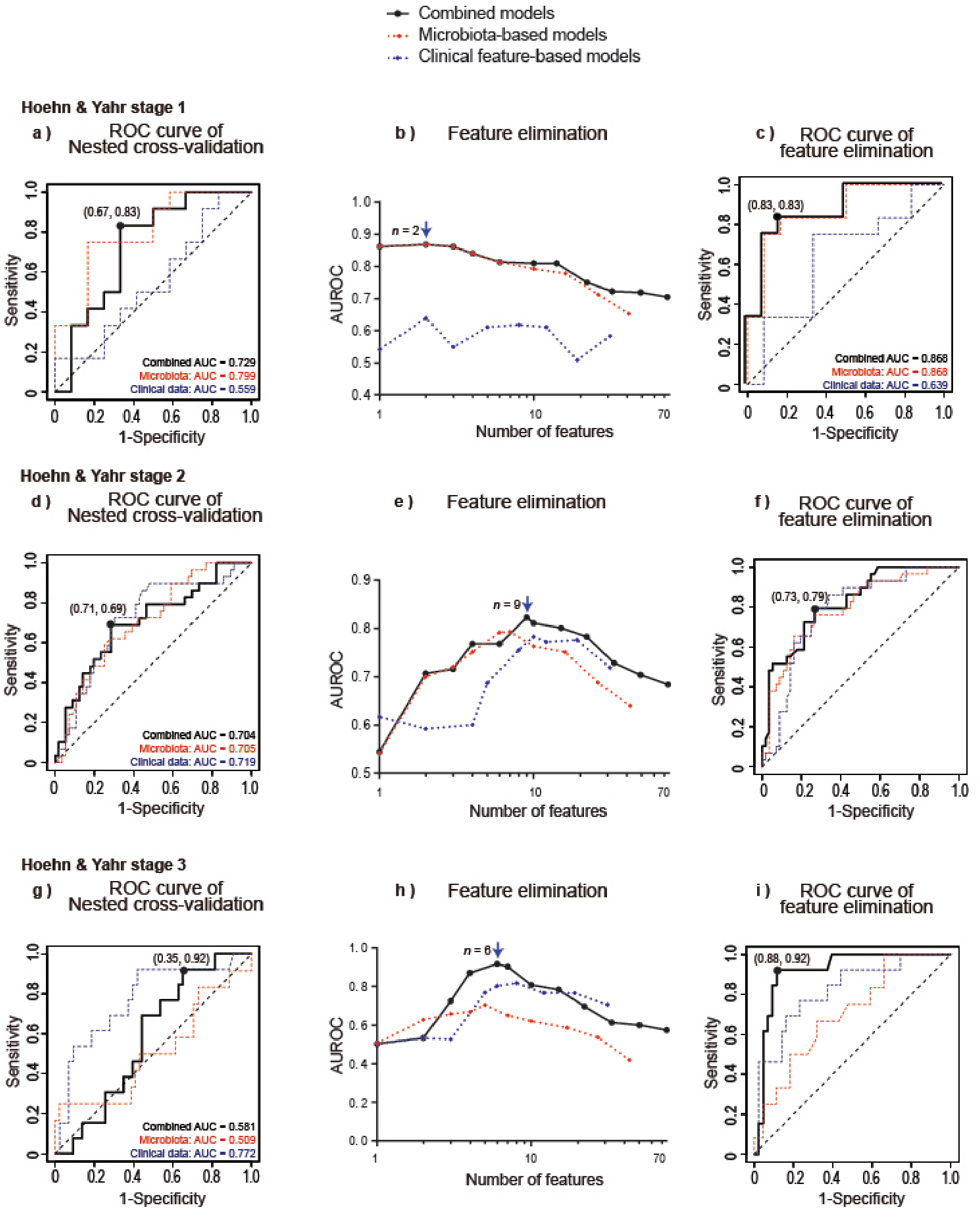

Supplementary Figure S1. Validation of modeling strategy by nested cross-validation and determination of essential features by cross-validation in combined models. (a, d, and g) ROC curves of nested cross-validation of random forest models for HY stages 1 **(a)**, 2 **(d)**, and 3 **(g)**, at year 0. Black solid lines represent combined models. The optimal point by Youden index is indicated by a dot with the specificity and sensitivity in parentheses. Red and blue dotted lines represent models constructed by bacterial and clinical features, respectively. **(b, e, and h)** AUROCs by leave-one-out cross-validation of random forest models for HY stages 1 **(b)**, 2 (**e**), and 3 (**h**) at year 0, while features were recursively eliminated. An arrow points to the maximum AUROC with the number of features. **(c, f, and i)** ROC curves of leave-one-out cross-validation

of random forest models at the maximum AUROC for HY stages 1 **(c)**, 2 (**f**), and 3 (**i**) at year 0. The optimal point by Youden index is indicated by a dot with the specificity and sensitivity in parentheses.

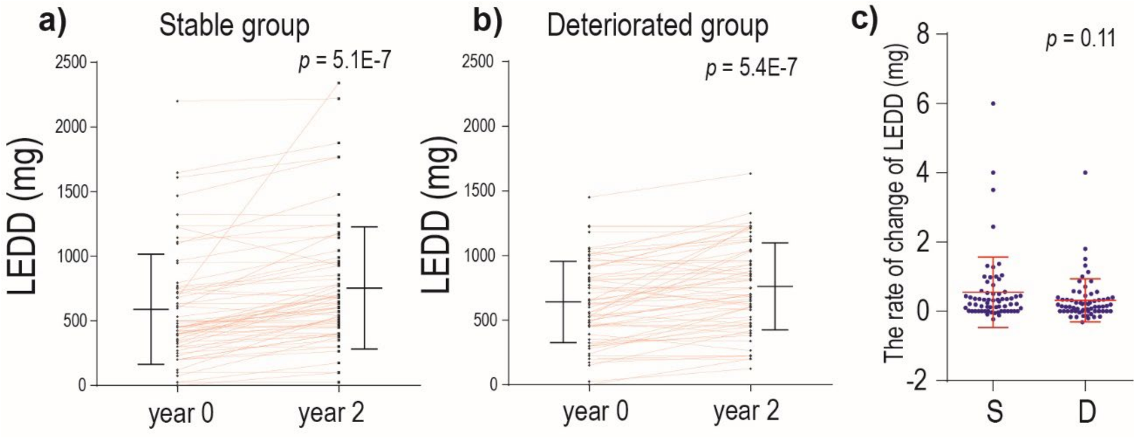

Supplementary Figure S2. a), b) The levodopa equivalent daily dosage (LEDD) at years 0 and 2 in the stable and deteriorated groups. Red lines connect LEDDs at years 0 and 2 in an identical patient. Mean and SD are indicated (*n* =72 for each of the stable and deteriorated groups). *P*-values are calculated by paired *t*-test.

c) The rates of changes of LEDD in two years in the stable (S) and deteriorated (D) groups. Mean and SD are indicated (*n* =72 for each of the stable and deteriorated groups). *P*-value is calculated by unpaired *t*-test.

Supplementary Table S1. Clinical and demographic features of PD patients at years 0 and 2

	Year 0[a]	Year 2[a]	*P*-value[b]
Age (years)	68.2 ± 8.6 (*n* = 224)	-	-
# Females	131 (*n* =224)	-	-
Body mass index (BMI)	21.6 ± 3.1 (*n* = 224)	21.4 ± 3.4 (*n* = 123)	0.47
# Constipation (≤ twice a week)	80 (*n* = 220)	46 (*n* = 125)	1
Stool frequency/week	4.7 ± 4.1 (*n* = 220)	4.3 ± 3.0 (*n* = 125)	0.36
Disease duration (years)	7.5 ± 6.1 (*n* = 224)	-	-
# Stool samples	224	113	-
Total MDS-UPDRS	49.7 ± 22.8 (*n* = 222) (range 11 to 153)	55.5 ± 26.8 (*n* = 146) (range 8 to 173)	*0.029
MDS-UPDRS III	26.6 ± 13.3 (*n* = 222) (range 4 to 84)	28.4 ± 16.1 (*n* = 146) (range 3 to 116)	0.26
Hoehn & Yahr scale	2.40 ± 0.87 (*n* = 224)	2.68 ± 0.93 (*n* = 182)	*2.4E-3
# Proton pump inhibitor	35 (*n* = 224)	13 (*n* = 144)	0.081
# H_2 blocker	9 (*n* = 224)	8 (*n* = 144)	0.61
# Antihyperlipidemic drug	37 (*n* = 224)	16 (*n* = 144)	0.17
# Angiotensin II receptor blocker	32(*n* = 224)	13 (*n* = 144)	0.15
# Calcium channel blocker	40 (*n* = 224)	21 (*n* = 144)	0.56
Levodopa/Carbidopa (mg)	351 ± 232 (*n* = 224)	459 ± 266 (*n* = 144)	*5.7E-5
# COMT inhibitor	73 (*n* = 224)	56 (*n* = 144)	0.22

# Anticholinergic agent	20 (*n* = 224)	12 (*n* = 144)	1.00
# Dopamine agonist	152 (*n* = 224)	100 (*n* = 144)	0.82
# MAO-B inhibitor	66 (*n* = 224)	46 (*n* = 144)	0.64
# Amantadine	36 (*n* = 224)	30 (*n* = 144)	0.27
# Smoking (never, past, present)	183 (82%), 32 (14%), 9 (4.0%)	99 (83%), 16 (13%), 5 (4.2%)	0.97
# Coffee (none, 1 or 2/week, 3 ~ 5/week, 6 or 7/week)	46 (21%), 51 (23%), 31 (14%), 96 (43%)	19 (16%), 20 (16%), 21 (17%), 62 (62%)	0.24
Walking and balance (MDS-UPDRS 2.12)	1.31 ± 1.03 (*n* = 222) (range 0 to 4)	1.63 ± 1.14 (*n* = 144) (range 0 to 4)	*7.5E-3
Freezing (MDS-UPDRS 2.13)	0.897 ± 1.03 (*n* = 222) (range 0 to 4)	1.16 ± 1.13 (*n* = 144) (range 0 to 4)	*0.025
Gait (MDS-UPDRS 3.10)	1.27 ± 1.02 (*n* = 222) (range 0 to 4)	1.51 ± 1.16 (*n* = 144) (range 0 to 4)	*0.038
Freezing of gait (MDS-UPDRS 3.11)	0.466 ± 0.902 (*n* = 222) (range 0 to 4)	0.683 ± 1.08 (*n* = 144) (range 0 to 4)	*0.040
Postural stability (MDS-UPDRS 3.12)	1.29 ± 1.31 (*n* = 222) (range 0 to 4)	1.63 ± 1.45 (*n* = 144) (range 0 to 4)	*0.022
Tremor (MDS-UPDRS 2.10)	0.874 ± 0.816 (*n* = 222) (range 0 to 3)	0.935 ± 0.809 (*n* = 144) (range 0 to 3)	0.5
Postural tremor of the hands (MDS-UPDRS 3.15)	0.910 ± 1.06 (*n* = 222) (range 0 to 6)	0.901 ± 1.06 (*n* = 144) (range 0 to 4)	0.94
Kinetic tremor of the hands (MDS-UPDRS 3.16)	1.03 ± 1.27 (*n* = 222)	0.845 ± 1.10 (n = 144)	0.15

	(range 0 to 6)	(range 0 to 4)	
Rest tremor of the hands (MDS-UPDRS 3.17)	0.520 ± 1.09 (*n* = 223) (range 0 to 6)	0.641 ± 1.24 (*n* = 144) (range 0 to 6)	0.33
Rest tremor of the legs (MDS-UPDRS 3.17)	0.229 ± 0.767 (*n* = 222) (range 0 to 5)	0.211 ± 0.690 (*n* = 144) (range 0 to 4)	0.83
Rest tremor of the lip/jaw (MDS-UPDRS 3.17)	0.0448 ± 0.311 (*n* = 222) (range 0 to 3)	0.0352 ± 0.249 (*n* = 144) (range 0 to 2)	0.76
Constancy of rest tremor (MDS-UPDRS 3.18)	0.574 ± 1.07 (*n* = 222) (range 0 to 4)	0.641 ± 1.17 (*n* = 144) (range 0 to 4)	0.58
MMSE	28.0 ± 2.6 (*n* = 223) (range 15 to 30)	28.3 ± 2.3 (*n* = 142) (range 14 to 30)	0.33

[a]Mean and SD are indicated when applicable. [b]Either Student's *t*-test or Fisher's exact test is applied. $^{*}P < 0.05$.

Supplementary Table S2. The differences of clinical features in the stable and deteriorated groups for Hoehn & Yahr stages 1, 2, and 3.

	Stable (S) vs Deteriorated (D)	
	P-value	Increased in S or D
**Hoehn & Yahr 1 at year 0**		
Sex	[b]0.32	S
Age	[a]0.67	D
BMI	[a]0.41	D
Stool frequency	[a]0.51	S
Disease duration	[a]0.14	D
Levodopa dosage	[a]0.28	D
COMT inhibitor	[b]1.00	n.a.
Anticholinergic agent	[b]1.00	n.a.
Dopamine agonist	[b]1.00	n.a.
MAO-B inhibitor	[b]1.00	n.a.
Amantadine	[b]1.00	n.a.
Proton pump inhibitor	[b]0.48	D
H_2 blocker	[b]1.00	n.a.
Antihyperlipidemic drug	[b]1.00	n.a.
Angiotensin II receptor blocker	[b]1.00	n.a.
Calcium channel blocker	[b]1.00	n.a.
Smoking	[b]0.48	D
Coffee	[b]1.00	n.a.
Walking and balance (MDS-UPDRS 2.12)	[a]0.67	S
Freezing (MDS-UPDRS 2.13)	[a]0.30	S
Gait (MDS-UPDRS 3.10)	[a]1.00	n.a.
Freezing of gait (MDS-UPDRS 3.11)	[a]1.00	n.a.
Postural stability (MDS-UPDRS 3.12)	[a]0.10	S
Tremor (MDS-UPDRS 2.10)	[a]0.20	D
Postural tremor of the hands (MDS-UPDRS 3.15)	[a]0.12	S
Kinetic tremor of the hands (MDS-UPDRS 3.16)	[a]0.38	S
Rest tremor of the hands (MDS-UPDRS 3.17)	[a]0.14	D
Rest tremor of the legs (MDS-UPDRS 3.17)	[a]0.65	D

Rest tremor of the lip/jaw (MDS-UPDRS 3.17)	[a]1.00	n.a.
Constancy of rest tremor (MDS-UPDRS 3.18)	[a]0.60	D
MMSE	[a]0.51	S
**Hoehn & Yahr 2 at year 0**		
Sex	[b]0.11	S
Age	[a]0.90	D
BMI	[a] 0.49	S
Stool frequency	[a] 0.66	D
Disease duration	[a]*3.3E-3	D
Levodopa dosage	[a]*0.024	D
COMT inhibitor	[b]*3.0E-4	D
Anticholinergic agent	[b]0.057	D
Dopamine agonist	[b]0.091	D
MAO-B inhibitor	[b]0.26	D
Amantadine	[b]*0.019	D
Proton pump inhibitor	[b]1.00	n.a.
H_2 blocker	[b]1.00	n.a.
Antihyperlipidemic drug	[b]0.15	S
Angiotensin II receptor blocker	[b]0.48	D
Calcium channel blocker	[b]0.76	S
Smoking	[b]0.90	D
Coffee	[b]0.56	S
Walking and balance (MDS-UPDRS 2.12)	[a]*0.047	D
Freezing (MDS-UPDRS 2.13)	[a]*0.021	D
Gait (MDS-UPDRS 3.10)	[a]*0.025	D
Freezing of gait (MDS-UPDRS 3.11)	[a]*8.4E-3	D
Postural stability (MDS-UPDRS 3.12)	[a]0.22	D
Tremor (MDS-UPDRS 2.10)	[a]0.10	S
Postural tremor of the hands (MDS-UPDRS 3.15)	[a]0.21	D
Kinetic tremor of the hands (MDS-UPDRS 3.16)	[a]*4.3E-3	D
Rest tremor of the hands (MDS-UPDRS 3.17)	[a]1.00	n.a.
Rest tremor of the legs (MDS-UPDRS 3.17)	[a]0.054	S
Rest tremor of the lip/jaw (MDS-UPDRS 3.17)	[a]0.17	D
Constancy of rest tremor (MDS-UPDRS 3.18)	[a]0.69	S

MMSE	0.13	S
**Hoehn & Yahr 3 at year 0**		
Sex	[b]1.00	n.a.
Age	[a]0.11	D
BMI	[a]0.22	S
Stool frequency	[a]*0.032	S
Disease duration	[a]0.13	D
Levodopa dosage	[a]0.32	D
COMT inhibitor	[b]0.055	D
Anticholinergic agent	[b]0.56	S
Dopamine agonist	[b]0.50	D
MAO-B inhibitor	[b]1.00	n.a.
Amantadine	[b]0.49	D
Proton pump inhibitor	[b]0.33	D
H_2 blocker	[b]1.00	n.a.
Antihyperlipidemic drug	[b]1.00	n.a.
Angiotensin II receptor blocker	[b]1.00	n.a.
Calcium channel blocker	[b]0.051	S
Smoking	[b]0.11	D
Coffee	[b]0.64	D
Walking and balance (MDS-UPDRS 2.12)	[a]*1.1E-3	D
Freezing (MDS-UPDRS 2.13)	[a]0.78	D
Gait (MDS-UPDRS 3.10)	[a]*1.3E-4	D
Freezing of gait (MDS-UPDRS 3.11)	[a]*2.3E-3	D
Postural stability (MDS-UPDRS 3.12)	[a]*1.3E-4	D
Tremor (MDS-UPDRS 2.10)	[a]0.33	D
Postural tremor of the hands (MDS-UPDRS 3.15)	[a]0.40	D
Kinetic tremor of the hands (MDS-UPDRS 3.16)	[a]0.14	D
Rest tremor of the hands (MDS-UPDRS 3.17)	[a]0.94	S
Rest tremor of the legs (MDS-UPDRS 3.17)	[a]0.49	D
Rest tremor of the lip/jaw (MDS-UPDRS 3.17)	[a]0.14	D
Constancy of rest tremor (MDS-UPDRS 3.18)	[a]0.88	D
MMSE	[a]0.62	D

[a]Student t-tests were applied. [b]Fisher's exact tests were applied. *$P < 0.05$

n.a., not applicable because *p*-value = 1.00.

Supplementary Table S3. Genera and clinical features used to construct the best random forest models to differentiate the stable and deteriorated groups for Hoehn & Yahr stages 1, 2, and 3

	Stable (S) vs Deteriorated (D)		
	[d]Feature importance	*P*-value	Increased in S or D
Hoehn & Yahr 1 at year 0			
Fusicatenibacter	0.535	[a]*7.0E-4	S
Faecalibacterium	0.465	[a]*5.6E-3	S
Hoehn & Yahr 2 at year 0			
Lactobacillus	0.187	[a]*2.0E-4	D
Anaerostipes	0.165	[a]*5.6E-3	S
Clostridium_sensu_stricto_1	0.108	[a]*0.030	D
**Kinetic tremor of the hands (UPDRS 3.16)**	0.100	[b]*4.3E-3	D
Bifidobacterium	0.0987	[a]*0.047	D
Blautia	0.0974	[a]*5.6E-3	S
Ruminococcus gnavus group	0.0905	[a]*0.015	S
Ruminococcus	0.0825	a0.67	D
**COMT-inhibitor**	0.0712	[c]*3.0E-4	D
Hoehn & Yahr 3 at year 0			
Turicibacter	0.227	[a]0.67	D
**Disease duration**	0.178	[b]0.13	D
**Gait**	0.177	[b]*1.3E-4	D
Incertae Sedis	0.144	[a]*0.032	D
Romboutsia	0.141	[a]0.73	D
**Postural stability (UPDRS 3.12)**	0.133	[b]*1.3E-4	D

Bold letters indicate clinical features.

[a]*P*-value was calculated by Wilcoxon rank-sum test of relative taxonimc abundances between the stable and deteriorated groups.

[b]*P*-value was calculated by *t*-test between the stable and deteriorated groups.

[c]*P*-value was calculated by Fisher's exact test between the stable and deteriorated groups.

[d]Sum of feature importances becomes 1.000 for each model.

*$P < 0.05$.

Supplementary Table S4. Combinations of medications in the stable and deteriorated groups at years 0 and 2

		Levodopa only	Levodopa and any other medications	Dopamine agonists without levodopa	No antiparkinson agents	*P*-value
Year 0	Stable group	8	61	2	1	0.37
	Deteriorated group	7	56	5	4	
Year 2	Stable group	5	65	1	1	0.82
	Deteriorated group	7	64	1	0	

P-values are calculated by Fisher's exact test.

Supplementary Table S5. Genera used to construct the best random forest model to differentiate the stable and deterioriated groups for Hoehn & Yahr stages 1-3, 1, 2, and 3

	Stable (S) vs Deteriorated (D)			
	[c]Feature importance	[d]*P*-value	Increased in S or D	Increased in C or P[1]
**Overall Hoehn & Yahr 1-3 at year 0**				
Fusicatenibacter	0.163	*3.8E-3	S	[a]C
Bifidobacterium	0.158	0.17	D	P
Subdoligranulum	0.148	0.21	D	C
Anaerostipes	0.138	0.17	S	C
Ruminococcus gnavus group	0.134	0.14	S	C
Blautia	0.130	*0.038	S	[a]C
Faecalibacterium	0.128	*6.3E-3	S	[a,b]C
**Hoehn & Yahr 1 at year 0**				
Fusicatenibacter	0.535	*7.0E-4	S	[a]C
Faecalibacterium	0.465	*5.6E-3	S	[a,b]C
**Hoehn & Yahr 2 at year 0**				
Lactobacillus	0.165	*2.0E-4	D	P
Blautia	0.151	*5.6E-3	S	[a]C
Fusicatenibacter	0.133	*9.5E-3	S	[a]C
Anaerostipes	0.160	*0.011	S	C
Ruminococcus gnavus group	0.137	*0.015	S	C
Akkermansia	0.114	*0.021	D	[a,b]P
Bifidobacterium	0.139	*0.047	D	P
**Hoehn & Yahr 3 at year 0**				
Incertae Sedis	0.220	*0.032	D	P
Lachnospiraceae anonymous	0.208	0.17	S	C
Clostridium sensu stricto 1	0.184	0.24	D	P
Oscillospiraceae UCG-005	0.191	0.27	D	P
Turicibacter	0.197	0.67	D	P

[a]Four genera were significantly changed in PD in our dataset in our previous report.[1]

[b]Two genera were significantly changed in PD in meta-analysis of five countries in our previous report.[1]

[c]Feature importance represents the imortance of genus in a random forest model.

[d]Sum of feature importances becomes 1.000 for each model.

$^{*}P < 0.05$

Supplementary Table S6. Clinical features to contruct the best random forest model to differentiate the stable and deteriorated groups for Hoehn & Yahr stages 1-3, 1, 2, and 3

	Stable (S) vs Deteriorated (D)		
	[c]Feature importance	*P*-value	Increased in S or D
**Overall Hoehn & Yahr 1-3 at year 0**			
BMI	0.16	[a]0.42	S
Disease duration	0.14	[a]*0.021	D
Age	0.14	[a]0.60	D
Levodopa dosage	0.12	[a]0.26	D
Stool frequency	0.11	[a]0.52	S
Hoehn & Yahr stage	0.091	[a]*0.019	S
Kinetic tremor of the hands (UPDRS 3.16)	0.088	[a]*0.023	D
Postural stability (UPDRS 3.12)	0.056	[a]0.54	D
Tremor (UPDRS 2.10)	0.049	[a]0.69	S
MMSE	0.047	[a]0.74	S
**Hoehn & Yahr 1 at year 0**			
BMI	0.52	[a]0.41	D
Age	0.48	[a]0.67	D
**Hoehn & Yahr 2 at year 0**			
COMT-inhibitor	0.14	[b]*3.0E-4	D
Disease duration	0.14	[a]*3.3E-3	D
Kinetic tremor of the hands (UPDRS 3.16)	0.18	[a]*4.3E-3	D
Levodopa dosage	0.11	[a]*0.024	D
Gait (UPDRS 3.10)	0.036	[a]*0.025	D
Tremor (UPDRS 2.10)	0.052	[a]*0.10	S
MMSE	0.042	[a]*0.13	D
BMI	0.13	[a]0.49	S
Stool frequency	0.11	[a]0.66	D
Age	0.070	[a]0.90	D
**Hoehn & Yahr 3 at year 0**			
Gait (UPDRS 3.10)	0.12	[a]*1.3E-4	D
Postural stability (UPDRS 3.12)	0.11	[a]*1.3E-4	D

Walking and balance (UPDRS 2.12)	0.097	[a*]1.1E-3	D
Stool frequency	0.12	[a*]0.032	S
Age	0.15	[a]0.11	D
Disease duration	0.14	[a]0.13	D
BMI	0.14	[a]0.22	S
Levodopa dosage	0.13	[a]0.32	D

Previously reported clinical features predictive of PD prognosis are underlined (see Discussion).[2-4] MMSE was a shared important feature for predicting the progression of total UPDRS.[5]

[a]*P*-value was calculated by *t*-test between the stable and deteriorated groups.

[b]*P*-value was calculated by Fisher's exact test between the stable and deteriorated groups.

[c]Sum of feature importances becomes 1.000 for each model.

[*]$P < 0.05$.

Supplementary references

1 Nishiwaki, H. *et al.* Meta-Analysis of Gut Dysbiosis in Parkinson's Disease. *Mov Disord,* doi:10.1002/mds.28119 (2020).

2 Post, B., Merkus, M. P., de Haan, R. J., Speelman, J. D. & Group, C. S. Prognostic factors for the progression of Parkinson's disease: a systematic review. *Mov Disord* **22**, 1839-1851; quiz 1988, doi:10.1002/mds.21537 (2007).

3 Oosterveld, L. P. *et al.* Prognostic factors for early mortality in Parkinson's disease. *Parkinsonism Relat Disord* **21**, 226-230, doi:10.1016/j.parkreldis.2014.12.011 (2015).

4 Macleod, A. D., Dalen, I., Tysnes, O. B., Larsen, J. P. & Counsell, C. E. Development and validation of prognostic survival models in newly diagnosed Parkinson's disease. *Mov Disord* **33**, 108-116, doi:10.1002/mds.27177 (2018).

5 Tsiouris, K. M., Konitsiotis, S., Koutsouris, D. D. & Fotiadis, D. I. Prognostic factors of Rapid symptoms progression in patients with newly diagnosed parkinson's disease. *Artif Intell Med* **103**, 101807, doi:10.1016/j.artmed.2020.101807 (2020).
